# Recent advances in the biosynthetic pathway and structural modification of gentamicin

**DOI:** 10.3389/fmicb.2025.1734985

**Published:** 2025-11-26

**Authors:** Xiaotang Chen, Ruifen Zou, Bao-Chen Qian, Xianpu Ni, Huanzhang Xia

**Affiliations:** 1 College of Medical Engineering, Jining Medical University, Jining, China; 2 School of Life Science and Biopharmaceutics, Shenyang Pharmaceutical University, Benxi, China

**Keywords:** gentamicin, biosynthesis, methylation network, transamination–epimerization network, di-deoxygenation, structural modification

## Abstract

As a member of the first-generation aminoglycoside antibiotics, gentamicin was once widely used in clinical practice. However, due to its drug resistance, toxicity, and side effects and the development of novel antibiotics, gentamicin has gradually faded from the spotlight. However, in recent years, aminoglycoside antibiotics have regained significant attention from the medical community as a critical therapeutic option for severe infections caused by multidrug-resistant bacteria. This review provides a detailed overview of the complete biosynthetic pathway of gentamicin and its recent advances, including the biosynthesis of the core structure 2-deoxystreptamine (2-DOS), the biosynthesis of the first pseudotrisaccharide intermediate gentamicin A2, the key branch point intermediate X2, and its parallel pathways. The methylation network, the transamination–epimerization network, and the di-deoxygenation processes within the gentamicin biosynthetic system are highlighted. Recent progress in the engineering of the gentamicin biosynthetic pathway and the development of novel gentamicin derivatives as new aminoglycoside antibiotics are also summarized. Finally, this review offers perspectives on the future research directions and uses of gentamicin. In summary, a detailed dissection of the complex biosynthetic network of gentamicin is provided to lay the groundwork for targeted and random structural modifications of gentamicin using combinatorial biosynthetic and high-throughput screening technologies to develop new aminoglycoside antibiotics with lower host toxicity and greater activity against resistant strains.

## 1 Introduction

**Figure 1:**
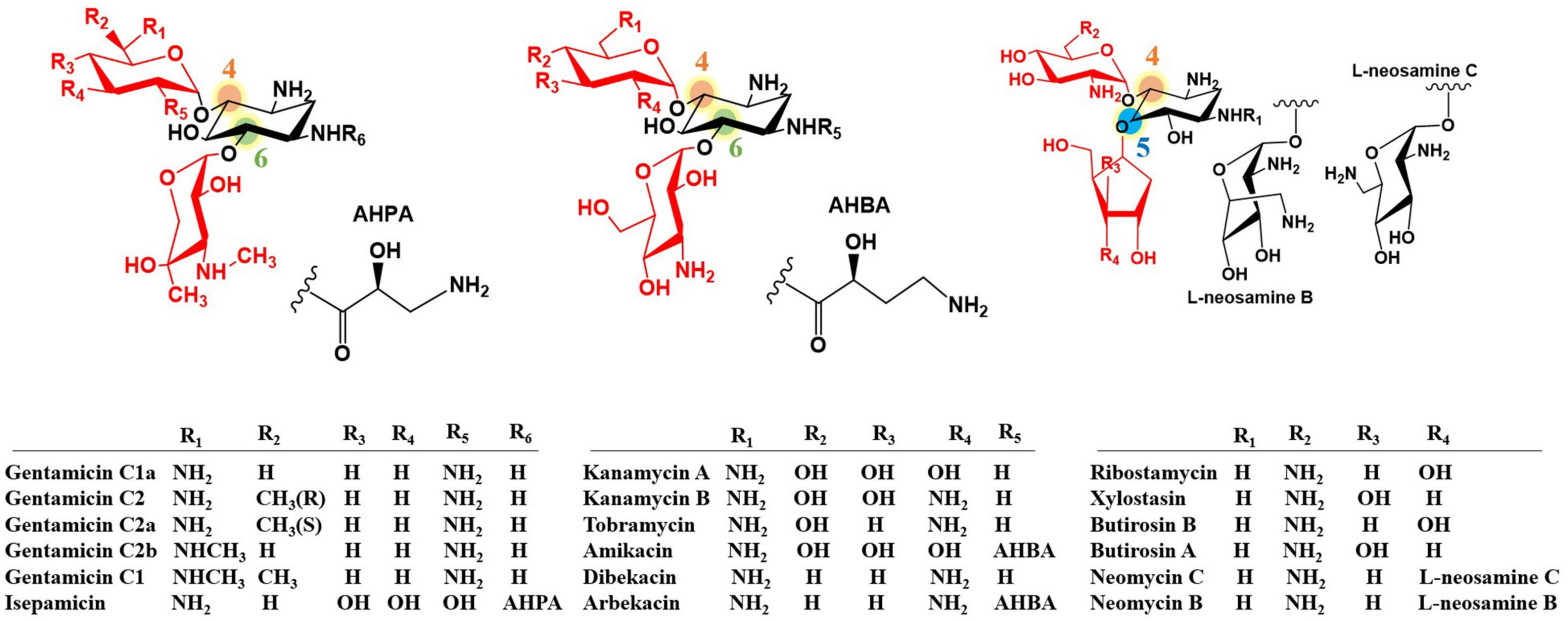
Structures of natural and semi-synthetic 2-DOS-containing aminoglycoside antibiotics.

Aminoglycoside antibiotics represent one of the oldest classes of antibiotics. They are characterized by two or three specific amino sugars linked via glycosidic bonds to an amino-substituted cyclohexane amino cyclitol. The first discovered aminoglycoside antibiotic, streptomycin, has streptidine as its core amino cyclitol structure. In contrast, other clinically valuable aminoglycosides are based on the core structure of 2-Deoxystreptamine (2-DOS) (Jones et al., 1944; Park et al., 2017; Busscher et al., 2005; Park et al., 2013; Kudo and Eguchi, 2016). 2-DOS-containing aminoglycosides are substituted at positions C-4 and C-5 or at C-4 and C-6 by amino sugars to form either 4,5-disubstituted aminoglycosides (such as butirosin and neomycin) or 4,6-disubstituted aminoglycosides (such as gentamicin, kanamycin, and tobramycin) (Figure 1).

Aminoglycoside antibiotics exert their bactericidal effect by binding to the 30S ribosomal subunit, thereby disrupting protein synthesis in bacteria (Magnet and Blanchard, 2005; Becker and Cooper, 2013; Poehlsgaard and Douthwaite, 2005). They have also shown potential for treating human immunodeficiency virus (HIV) and certain human genetic disorders (Zingman et al., 2007; Prayle and Smyth, 2010; Stelzer et al., 2011; Ennifar et al., 2013). The widespread antibiotic resistance to aminoglycosides has significantly limited their clinical utility and poses a serious clinical challenge (Magnet and Blanchard, 2005; Becker and Cooper, 2013). The most common resistance mechanism involves aminoglycoside inactivation by aminoglycoside-modifying enzymes (AMEs) (Ramirez and Tolmasky, 2010). Fortunately, certain 2-DOS-containing aminoglycosides have intrinsic structural features that confer resistance to AMEs. These features include the *N*-acyl side chain of butirosin (including the 4-amino-2-hydroxybutyrate substitution at the C-1 amino group of the 2-DOS core), C-3′ and C-4′ di-deoxygenation of gentamicin, and C-3′ deoxygenation of tobramycin. These naturally occurring structural motifs have inspired the development of second-generation semi-synthetic aminoglycoside antibiotics, including amikacin, dibekacin, and arbekacin (Figure 1). Therefore, structural modification of aminoglycosides is crucial for enhancing their efficacy and reducing their toxicity (Fosso et al., 2015; Zhou et al., 2007; Poulikakos and Falagas, 2013). Although traditional chemical approaches have achieved some success (Sucheck and Shue, 2001; Shi et al., 2013), biotechnological strategies, particularly combinatorial biosynthesis, have emerged as more promising avenues for developing novel aminoglycoside antibiotics (Park et al., 2017; Winter and Tang, 2012; Kim et al., 2015; Butler et al., 2016; Pawlowski et al., 2016).

As a broad-spectrum aminoglycoside antibiotic, gentamicin plays a critical role in the treatment of Gram-negative bacterial infections. However, its therapeutic efficacy is increasingly challenged by the growing problem of bacterial resistance (Shaw et al., 1993). Research on biosynthesis is essential for the discovery and development of novel aminoglycoside analogs with enhanced therapeutic properties. For instance, glycosyltransferases in the kanamycin biosynthetic pathway exhibit broad substrate specificity, utilizing sugar donors derived from primary metabolism. Similarly, the post-modification systems in gentamicin biosynthesis demonstrate remarkable substrate flexibility. Therefore, by employing combinatorial biosynthesis strategies, the functional characteristics of glycosyltransferases and post-modification systems in gentamicin biosynthesis can be harnessed to rationally design and directionally assemble diverse structural units, thereby facilitating the development of novel aminoglycoside antibiotics (Xu et al., 2025). Our group has maintained a long-term research focus on aminoglycoside antibiotics, among which gentamicin serves as an exemplary representative. The recent elucidation of the gentamicin di-deoxygenation pathway has unveiled novel breakthroughs and research directions in the biosynthesis of gentamicin and its derivatives. This review aims to provide a comprehensive yet focused analysis specifically centered on gentamicin. Considering that the biosynthetic pathways of other aminoglycoside antibiotics have been well-reviewed (Kudo and Eguchi, 2016; Park et al., 2017; Yu et al., 2017; Li and Sun, 2019; Ban et al., 2020; Obszynski et al., 2022), we have strategically focused on gentamicin as a representative species to avoid unnecessary duplication in this review.

## 2 A comprehensive overview of gentamicin

**Figure 2:**
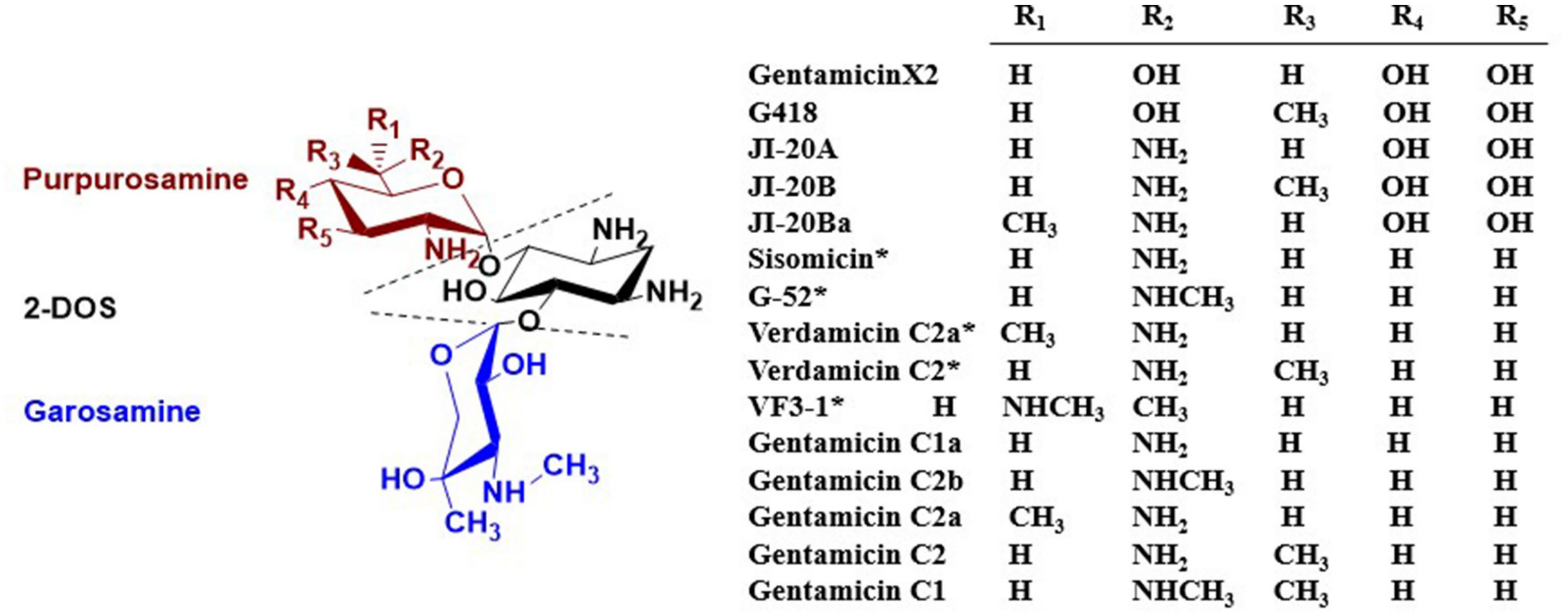
Structures of gentamicin components found in *M. echinospora* fermentation broth. *Indicates the presence of a C-4′, C-5′ double bond.

Gentamicin is a broad-spectrum, alkaline antibiotic complex first isolated by Weinstein et al. in 1963 from two strains of *Micromonospora* (Weinstein et al., 1963). It belongs to the 4,6-disubstituted-2-DOS class of aminoglycoside antibiotics, which inhibit protein synthesis in Gram-negative bacteria. Its structure consists of the core 2-DOS moiety linked via glycosidic bonds at the C-4 and C-6 positions to purpurosamine and garosamine, respectively (Park et al., 2013). The primary products of wild-type *M. echinospora* fermentation are the gentamicin C complex, with the major components C1, C1a, C2, and C2a, and trace amounts of C2b (Lee et al., 1976; Daniels et al., 1975). Gentamicin biosynthesis involves multiple complex enzymatic reactions; therefore, in addition to the main C components, the fermentation broth also contains minor secondary metabolites. These include compounds with C-3′, C-4′ hydroxylated structures, such as gentamicin A2, X2, G418, JI-20A, JI-20B, and JI-20Ba (Odakura et al., 1983; Gu et al., 2015), and structures featuring a C-4′, C-5′ double bond, including sisomicin (Bérdy et al., 1977), 6′-*N*-methylsisomicin (G-52) (Marquez et al., 1976), verdamicin C2a, verdamicin C2 (Weinstein et al., 1975; Hanessian et al., 2009), and 6′-*N*-methylverdamicin (VF3-1) (Kase et al., 2006). The structures of these components are illustrated in Figure 2. Park et al. established a rapid, sensitive, and highly selective analytical method based on high-performance liquid chromatography coupled with electrospray ionization mass spectrometry (HPLC-ESI-MS) to profile biosynthetic intermediates in the gentamicin pathway of *Micromonospora echinospora*. This approach provides an efficient tool for elucidating the gentamicin biosynthetic pathway and supporting related metabolic engineering studies (Park et al., 2007).

## 3 Gentamicin biosynthetic pathway

**Figure 3:**
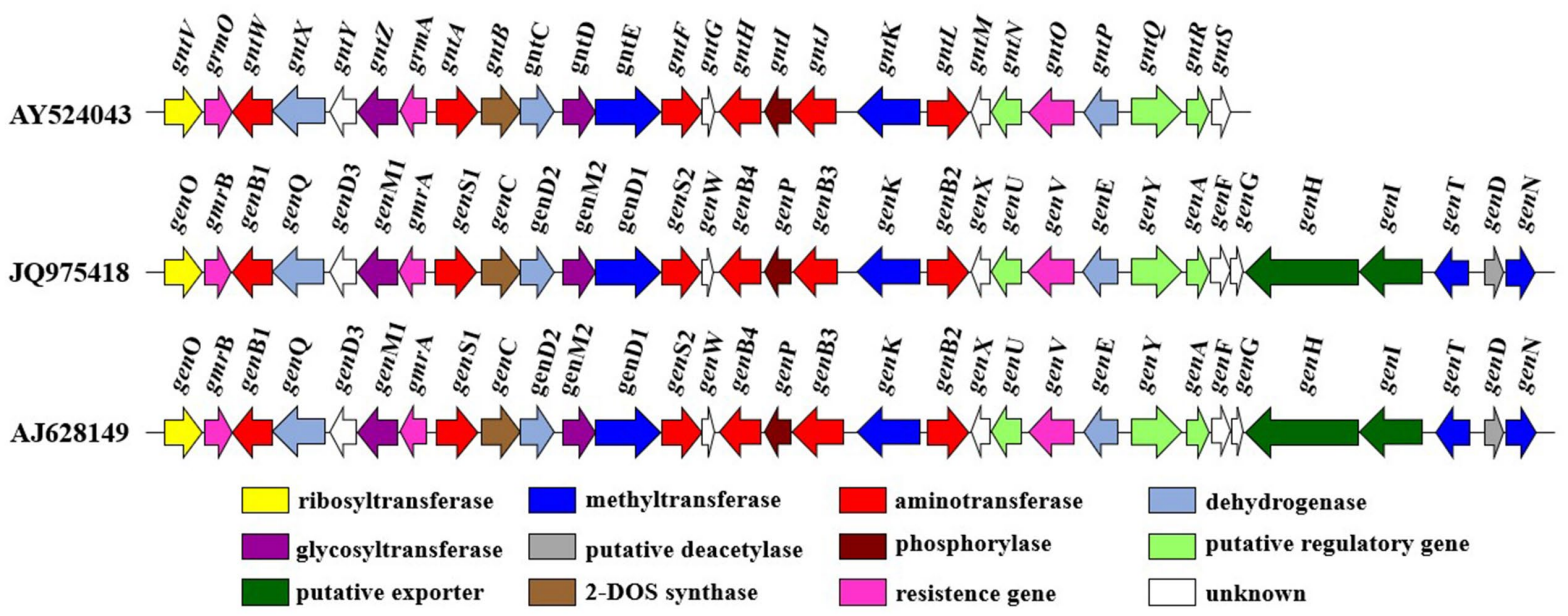
The gentamicin biosynthetic gene cluster.

The gentamicin biosynthetic pathway was initially proposed by Testa and Tilley by studying a paromamine-producing mutant strain of *M. echinospora*. They observed that gentamicin C1 was not converted into other compounds, whereas gentamicin C2 was transformed into C1, and gentamicin C1a was converted into C2b (Testa and Tilley, 1976). Advances in modern DNA sequencing technologies and molecular biology have since ushered in a new era for elucidating the gentamicin biosynthetic pathway. Biosynthetic gene clusters of gentamicin from different strains have been fully sequenced (GenBank Accession No. AY524043, JQ975418, AJ628149) (Figure 3) (Unwin et al., 2004). By applying bioinformatics to perform sequence alignment and predict the function of individual genes, and by constructing biosynthetic gene knockout mutants combined with heterologous expression experiments, the functions of most genes involved in gentamicin biosynthesis have been elucidated. As a result, the gentamicin biosynthetic pathway has been largely deciphered.

### 3.1 Biosynthesis of the gentamicin core structure, 2-DOS

**Figure 4:**
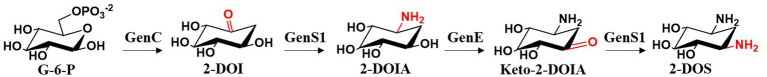
The biosynthetic pathway of 2-DOS.

Gentamicin biosynthesis begins with D-glucose (Rinehart et al., 1974). Initially, the C-6 position of D-glucose is phosphorylated to form D-glucose-6-phosphate (G-6-P). This is followed with catalysis by 2-deoxy-*scyllo*-inosose synthase (2-DOI synthase, GenC), which converts D-glucose-6-phosphate into 2-deoxy-*scyllo*-inosose (2-DOI). Subsequently, a transamination reaction occurs at the C-1 position, mediated by 2-deoxy-*scyllo*-inosose aminotransferase (GenS1), to 2-deoxy-*scyllo*-inosamine (2-DOIA) (Tamegai et al., 2002). Next, dehydrogenation at the C-3 position is mediated by GenE, forming 3-keto-2-deoxy-*scyllo*-inosamine (3-keto-2-DOIA). Finally, a second catalytic step by the 2-deoxy-*scyllo*-inosose aminotransferase GenS1 introduces an amino group at the C-3 position, ultimately forming the core structure of gentamicin, 2-deoxystreptamine (2-DOS) (Figure 4) (Huang et al., 2002; Kudo et al., 2005).

### 3.2 Biosynthesis of the first pseudotrisaccharide intermediate, gentamicin A2

**Figure 5:**
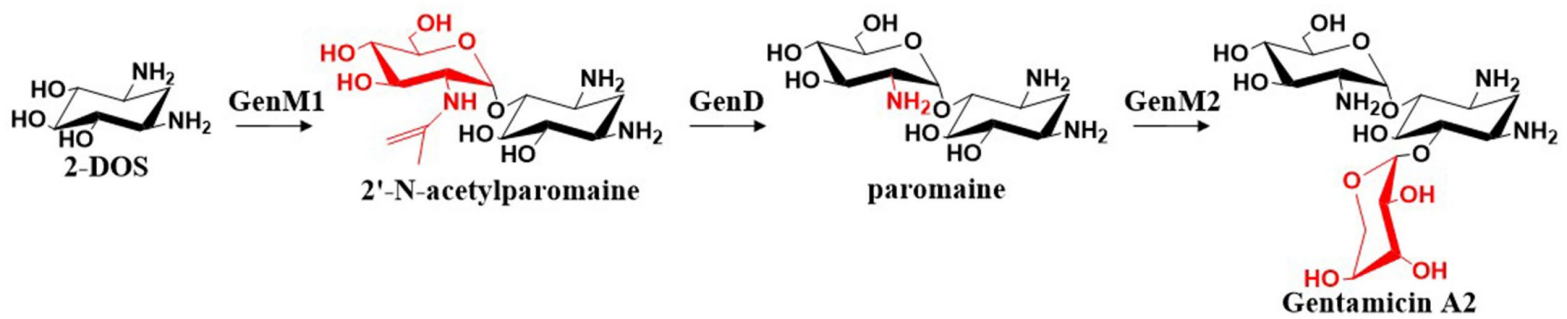
The biosynthetic pathway of gentamicin A2.

Park et al. (2008) cloned the gentamicin biosynthetic genes *gen*C, *gen*S1, *gen*E, *gen*M1, *gen*D, and *gen*M2 and heterologously expressed them in *Streptomyces venezuelae* YJ003 to elucidate the biosynthetic pathway from G-6-P to the first pseudotrisaccharide intermediate, gentamicin A2. *Gen*M1 was found to encode a 4-*O*-glycosyltransferase that catalyzes the glycosidic linkage of UDP-*N*-acetyl-D-glucosamine (UDP-GlcNAc) to the C-4 position of 2-DOS, yielding 2′-*N*-acetylparomamine. This intermediate is then deacetylated by GenD to form paromamine. *Gen*M2 encodes the 6-*O*-glycosyltransferase that attaches UDP-xylose to the C-6 position of 2-DOS through a glycosidic bond to form gentamicin A2. The overall biosynthetic process is illustrated in Figure 5.

### 3.3 The key branch point intermediate X2 and its parallel pathways in gentamicin biosynthesis

**Figure 6:**
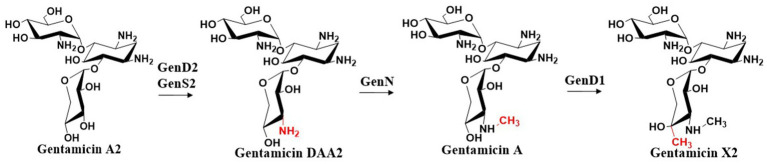
The biosynthetic pathway of gentamicin X2.

Gentamicin A2 is converted to gentamicin X2 through a series of enzymatic reactions. The dehydrogenase GenD2 and aminotransferase GenS2 are responsible for amination at the C-3′′ position of gentamicin A2. The resulting amino group is then methylated by the *S*-adenosylmethionine (SAM)-dependent *N*-methyltransferase GenN to form a C-3′′-aminomethyl group (Huang et al., 2015). GenD1, a class B radical SAM-dependent methyltransferase (Zhang et al., 2012; Bauerle et al., 2015), incorporates a methyl group at the C-4′′ position using cobalamin as a cofactor, ultimately yielding gentamicin X2 (Ding et al., 2016; Zhou et al., 2016) (Figure 6). Sun et al. systematically elucidated the biotransformation process from the initial trisaccharide compound gentamicin A2 to the key branch point intermediate gentamicin X2 through integrated molecular genetics and biochemical approaches (Huang et al., 2015). By constructing in-frame deletions to inactivate four critical genes in this pathway—*gen*D2, *gen*S2, *gen*N, and *gen*D1—they subsequently fed intermediate metabolites to the mutant strains and predicted the functional correspondence between target genes and catalytic steps based on alterations in fermentation products. The Dias’s research group elucidated the crystal structure of methyltransferase GenN, confirming its methylation activity toward a series of substrates (Bury et al., 2017). Analysis of its three-dimensional structure revealed the structural basis for its broad substrate specificity. The group also determined the structure of GenD2, another key enzyme in gentamicin biosynthesis, which functions as an NAD+/NADP+-dependent oxidoreductase responsible for C-3′’ dehydrogenation (De Araújo et al., 2019). Structural analysis uncovered two distinct β-sheet domains and their roles in dimer formation and substrate recognition.

**Figure 7:**
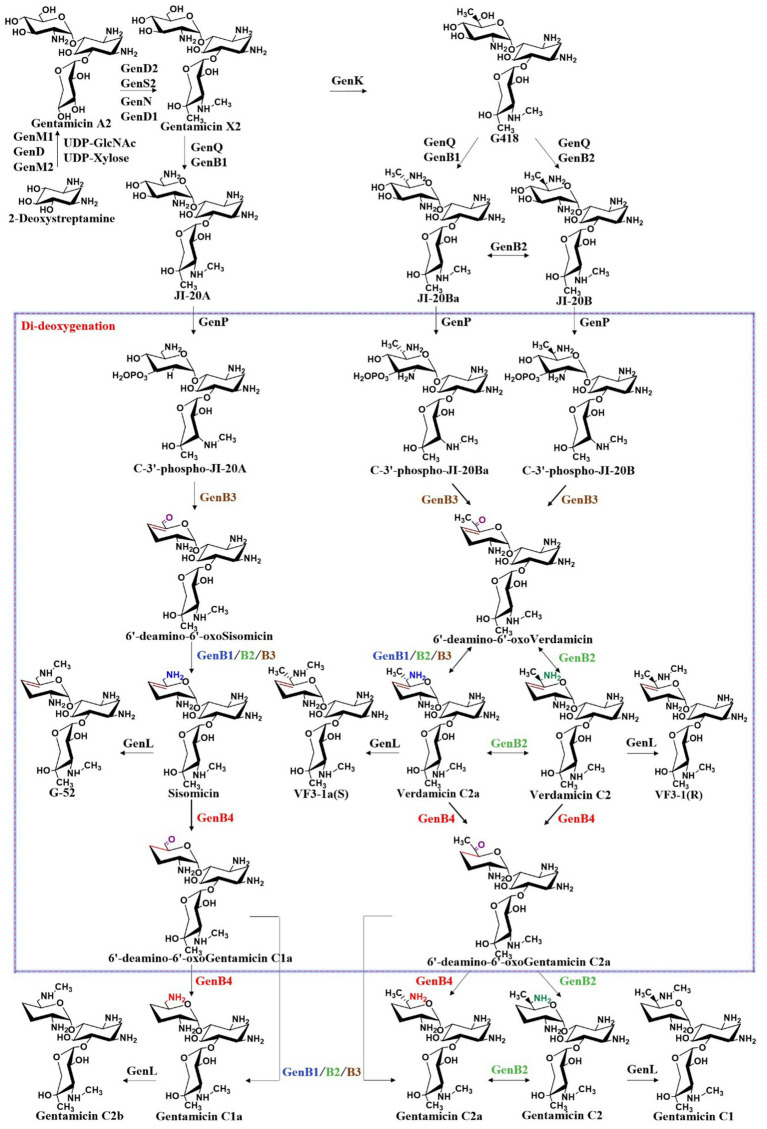
The biosynthetic pathway of gentamicin.

From the key intermediate gentamicin X2, the pathway diverges in parallel, leading to the formation of JI-20A, JI-20Ba, and JI-20B, which subsequently give rise to gentamicins C1a, C2b, C2, C2a, and C1. Similar to GenD1, GenK is also a class B radical SAM-dependent methyltransferase. GenK-mediated methylation directs the biosynthetic pathway toward the production of gentamicins C2, C2a, and C1, whereas the absence of this methylation leads to the formation of gentamicins C1a and C2b. The identification of this branch point provides crucial insights into the regulation of gentamicin biosynthesis. Furthermore, *in vitro* studies of GenK activity have elucidated its catalytic mechanism, demonstrating the transfer of the *S*-methyl group from SAM to cobalamin and subsequently to the aminoglycoside substrate, accompanied by the generation of 5′-deoxyadenosine and *S*-adenosylhomocysteine (Kim et al., 2013). Through experiments employing deuterated substrates, this group also demonstrated that the 6′-pro-R hydrogen atom of gentamicin X2 is stereoselectively abstracted by the 5′-dAdo• radical, and methylation at the C6′ position proceeds with retention of configuration (Kim et al., 2017). It catalyzes methylation at the C-6′ position of gentamicin X2, yielding G418, a key intermediate in the parallel biosynthetic pathway (Kim et al., 2013). G418 and gentamicin X2 are the starting substrates for their respective parallel pathways. G418 is sequentially modified by the dehydrogenase GenQ and the aminotransferase GenB1 or GenB2 to form JI-20Ba or JI-20B, respectively, while GenQ and GenB1 catalyze the intermediate J1-20A from gentamicin X2 (Figure 7). JI-20A is then converted into gentamicin C1a through the action of a C-3′, C-4′ dideoxygenase system. Subsequently, the *N*-methyltransferase GenL introduces a methyl group at the C-6′ amino group of C1a to generate gentamicin C2b (Li et al., 2018). In the other parallel pathway, JI-20B and JI-20Ba are also processed by the dideoxygenase system to produce gentamicin C2 and gentamicin C2a. Finally, gentamicin C2 is further methylated at the C-6′ position by the *N*-methyltransferase GenL to yield gentamicin C1. GenL, responsible for the ultimate methylation step in gentamicin biosynthesis, was successfully identified by Li et al. through a combination of whole-genome sequencing, *in vitro* enzymology, and *in vivo* genetic engineering, thus closing the final gap in the gentamicin methylation network (Li et al., 2018). The complete gentamicin biosynthetic pathway is summarized in Figure 7.

### 3.4 Methylation network in the gentamicin biosynthetic system

**Figure 8:**
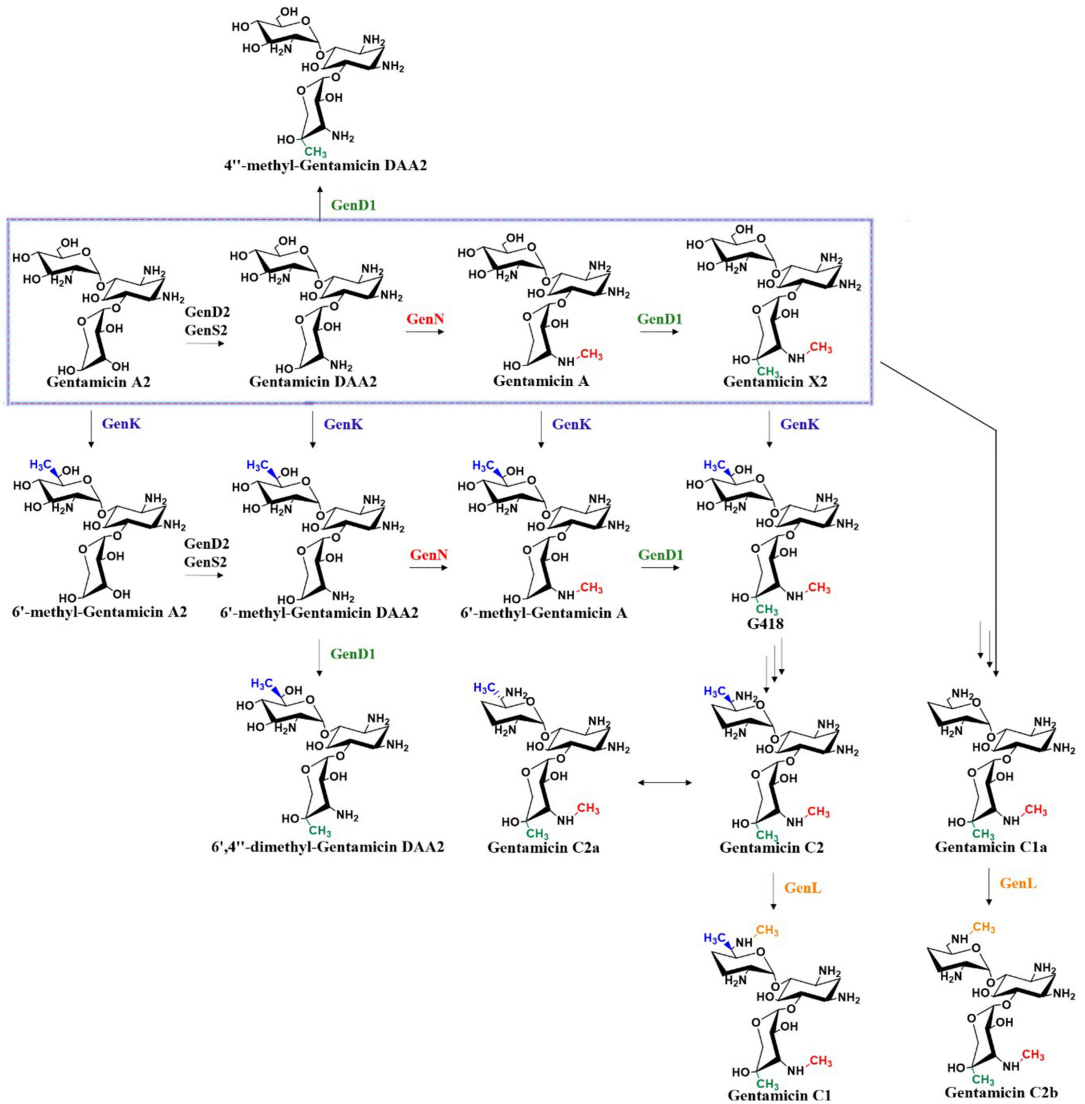
Methylation network in the gentamicin biosynthetic system.

The branching point in the gentamicin biosynthetic pathway is determined by the substrate selectivity of methyltransferases GenN, GenD1, and GenK, which collectively constitute a methylation network capable of ectopically modifying early-stage intermediates. Methylation is a crucial reaction in biosynthesis, as it can generate considerable chemical diversity in final products. In the late stage of gentamicin biosynthesis, four methyltransferases—the 3′′-*N*-methyltransferase GenN, 4′′-*C*-methyltransferase GenD1, 6′-*C*-methyltransferase GenK, and 6′-*N*-methyltransferase GenL—form an intricate methylation network. These four enzymes act on distinct positions, N-3′′, C-4′′, C-6′, and N-6′, respectively, to create parallel paths in a stereochemically defined methylation system (Figure 8) (Li et al., 2018). This network exhibits a certain degree of substrate flexibility while maintaining relative specificity. For example, GenK demonstrates the capacity to catalyze methylation at the C-6′ position of nearly all pseudotrisaccharide intermediates, yet exhibits catalytic incompetence toward both the conversion of 4″-methyl-gentamicin DAA2 to 6′,4″-dimethyl-gentamicin DAA2 and the C-6′ methylation of gentamicin C2b to yield gentamicin C1. These observations indicate that the presence of a methyl group at the C-4″ position or an aminomethyl group at the N-6′ position exerts inhibitory effects on GenK’s methyltransferase activity. Notably, GenK effectively catalyzes the C-6′ methylation of gentamicin X2 to form G418, suggesting that concurrent methylation at both the N-3″ and C-4″ positions alleviates such inhibition (Kim et al., 2013; Kim et al., 2017; Li et al., 2018). GenN catalyzes N-3″ methylation of both gentamicin DAA2 and 6′-methyl-gentamicin DAA2, but fails to modify 4″-methyl-gentamicin DAA2 or 6′,4″-dimethyl-gentamicin DAA2, indicating suppression of its activity by C-4″ methyl group. In contrast, the 4″-*C*-methyltransferase activity of GenD1 remains unaffected by the presence of N-3″ or C-6′ methyl groups (Li et al., 2018). Furthermore, GenL mediates N-6′ methylation of both gentamicin C2 and gentamicin C1a, yet displays no activity toward gentamicin C2a, the C-6′ epimer of gentamicin C2 (Li et al., 2018). The relatively strict selectivity of these four methyltransferases ensures the dominance of the canonical biosynthetic pathway in wild-type strains. Methyltransferase systems in natural product biosynthesis, such as this one, have great potential for modifying and developing novel derivatives.

### 3.5 Transamination–epimerization network in the gentamicin biosynthetic system

Within the gentamicin biosynthetic gene cluster, four pyridoxal phosphate (PLP)-dependent aminotransferases—GenB1, GenB2, GenB3, and GenB4—exhibit high sequence homology and demonstrate aminotransferase activity toward the C6′ position of pseudotrisaccharide intermediates in gentamicin biosynthesis (Guo et al., 2014). Among them, GenB2 additionally exhibits C-6′ epimerization activity (Gu et al., 2015), GenB3 possesses di-dehydroxylation activity (Zhou et al., 2021; Li et al., 2021), and GenB4 displays 4′,5′-double bond reduction activity (Chen et al., 2020). These enzymes play crucial roles in the modification of intermediates within the branched pathways and are essential for the di-deoxygenation process. Guo et al. demonstrated that all four enzymes can catalyze the transamination of 6′-deamino-6′-oxo-gentamicin X2 (6′-DOX) and 6′-deamino-6′-oxo-G418 (6′-DOG) to form JI-20A and JI-20B, respectively, with GenB1 exhibiting the highest catalytic activity (Guo et al., 2014). Gu et al. proposed that, in addition to its transaminase activity, GenB2 also exhibits C-6′ amino epimerase activity (Gu et al., 2015). G418 is converted into JI-20B through the action of dehydrogenase GenQ and the bifunctional transaminase and C-6′ epimerase GenB2, while it is transformed into JI-20Ba through the action of GenQ with any one of the transaminases GenB1, GenB3, or GenB4. GenB2 also catalyzes the epimerization between JI-20B and JI-20Ba, and between gentamicin C2 and C2a. In a significant structural study, Gabriel et al. determined the holo form of GenB2 and its complexes with two substrate analogs, G418 and gentamicin X2. They confirmed that GenB2 is a fold I-type PLP-dependent enzyme that catalyzes epimerization through a mechanism distinct from that of fold III PLP-dependent epimerases. Their findings provide valuable insights supporting the enzymatic production of novel aminoglycoside derivatives (Oliveira et al., 2024). As shown in Figure 7, the transamination process from 6′-deamino-6′-oxogentamicin C1a to gentamicin C1a was investigated using different amino donors and aminotransferases (GenB1, GenB2, GenB3, and GenB4). The results indicated that L-glutamate serves as the optimal amino donor, with GenB1 exhibiting the highest transaminase activity, followed by GenB2, while GenB3 and GenB4 demonstrated relatively weak transaminase activities (Chen et al., 2020). In a parallel pathway, GenB4 catalyzes the conversion of verdamicin C2a to gentamicin C2a, and the combined action of GenB4 and GenB2 catalyzes the formation of gentamicin C2 from verdamicin C2. However, neither GenB4 nor the GenB4–GenB2 system could catalyze the reduction of 6′-deamino-6′-oxoverdamicin, suggesting that the C-6′ amino group is essential for the reductase activity of GenB4. Furthermore, the transamination-deamination equilibrium among 6′-deamino-6′-oxoverdamicin, verdamicin C2a, and verdamicin C2 was found to favor deamination. This observation explains the significant accumulation of 6′-deamino-6′-oxoverdamicin in the *M. echinospora* △genB4 mutant (Chen et al., 2020). By integrating recent advances in the understanding of gentamicin di-deoxygenation mechanisms, it is evident that the biosynthetic system contains a complex transamination–epimerization network. Elucidating the substrate preferences of these bifunctional transaminase and epimerase enzymes and identifying the key residues governing their activity are of great importance for the rational construction of microbial strains that can generate high yields of specific gentamicin components, and for the development of new therapeutic agents.

## 4 C-3′, C-4′ di-deoxygenation process in gentamicin biosynthesis

### 4.1 Significance of the di-deoxygenation process

**Figure 9:**
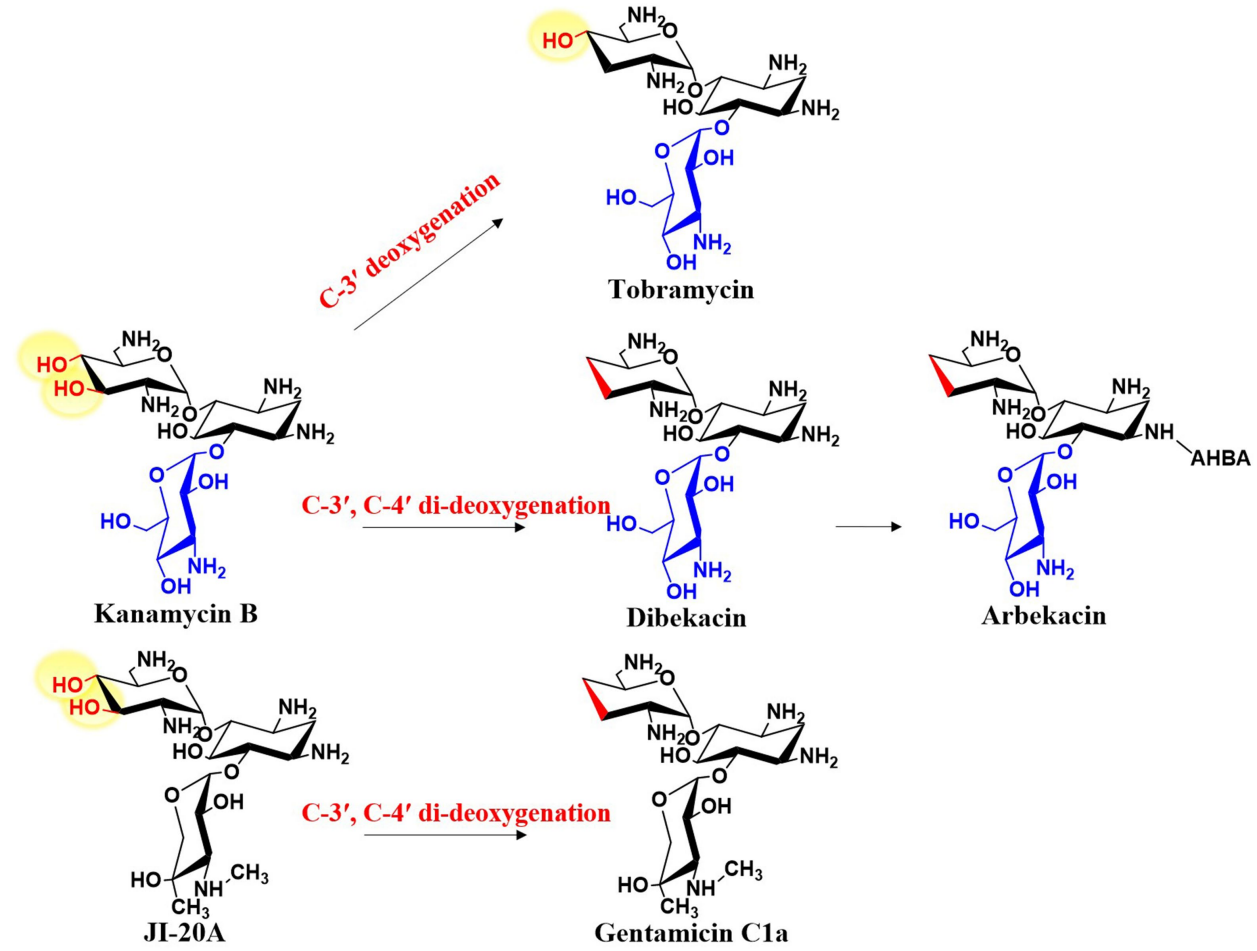
Deoxygenation modification of aminoglycoside antibiotics.

2-DOS-containing aminoglycoside antibiotics have intrinsic structural features that confer resistance to AMEs. One of the most common features is deoxygenation, as the hydroxyl groups in aminoglycosides are primary targets for inactivation by bacterial resistance enzymes. Based on the extent of deoxygenation, these antibiotics can be classified into two categories: those with C-3′ deoxygenation, including tobramycin, apramycin, and lividomycin, and those with C-3′ and C-4′ di-deoxygenation, including gentamicin, sisomicin, verdamicin, fortimycin, and istamycin. Deoxygenation significantly enhances the antibiotic efficacy of the molecule. For example, the only structural difference between kanamycin B and tobramycin is that kanamycin B has a hydroxyl group at the C-3′ position, whereas tobramycin has a deoxy structure at the same site (Figure 9). Notably, the efficacy of tobramycin is superior to that of kanamycin B, which is why tobramycin remains in clinical use. Removing oxygen atoms can prevent enzymatic modifications at these positions by bacterial enzymes while preserving antibiotic activity. Therefore, this modification strategy has been used to develop semi-synthetic antibiotics such as arbekacin and dibekacin (Figure 9). Interestingly, in aminoglycoside antibiotics, none of the deoxygenated hexose moieties are deoxygenated at the C-6 position. In contrast, all previously studied deoxyhexoses share C-6 deoxygenation, with any additional deoxygenations based on this C-6 deoxy structure (He and Liu, 2002). This suggests that gentamicin and related aminoglycosides are synthesized through a unique deoxygenation pathway distinct from the conventional route.

### 4.2 The catalytic process of di-deoxygenation

**Figure 10:**
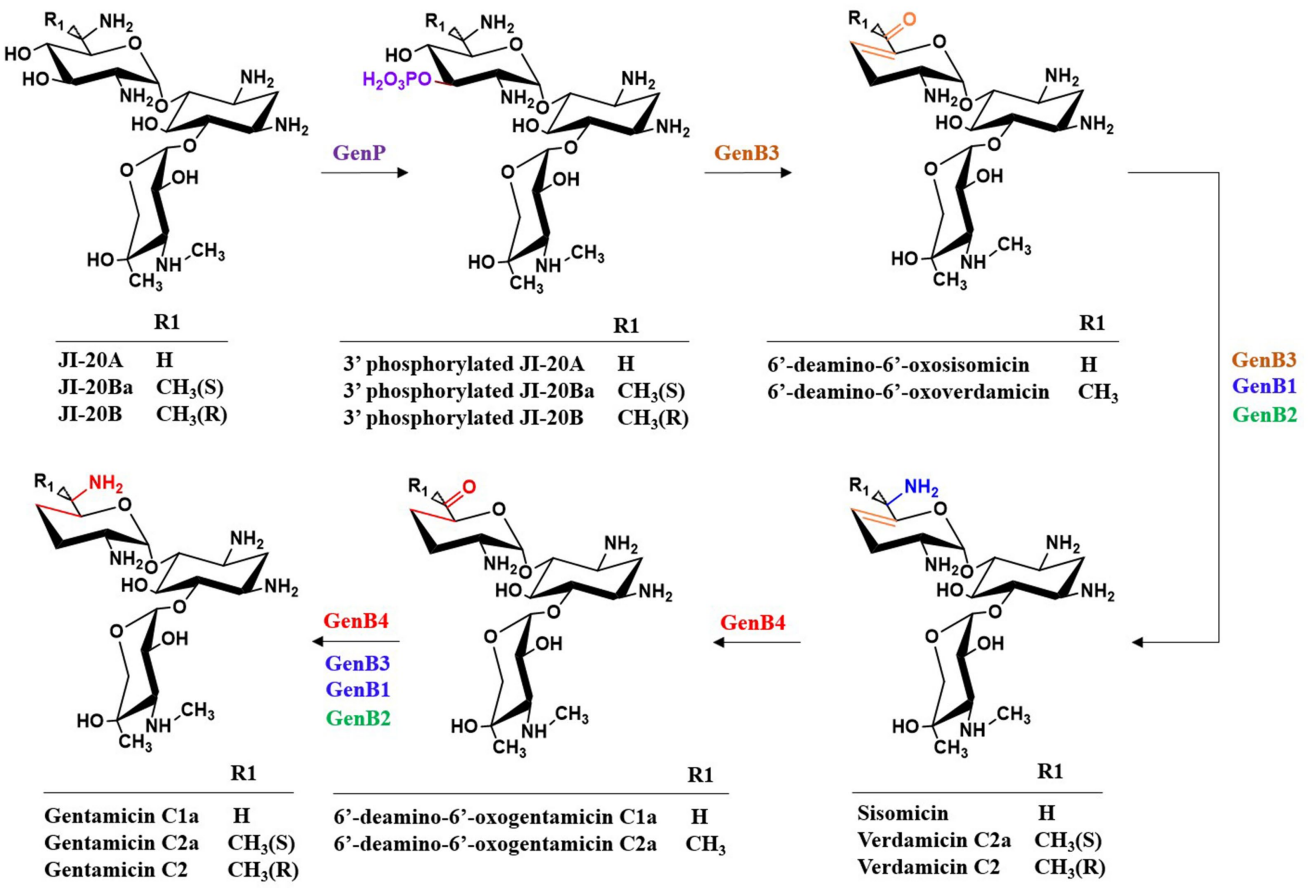
The catalytic process of di-deoxygenation in gentamicin biosynthesis.

The di-deoxygenation process is one of its most complex steps in gentamicin biosynthesis, and therefore has long been a research focus. After nearly a decade of investigation, this mechanism has finally been elucidated. GenP catalyzes the phosphorylation of the C-3′-OH group of JI-20A (as well as JI-20Ba and JI-20B in the parallel pathway), yielding C-3′-phospho-JI-20A (C-3′-phospho-JI-20Ba and C-3′-phospho-JI-20B, respectively), thereby initiating the first step of the di-deoxygenation reaction (Gu et al., 2015; Shao et al., 2013) (Figure 10). Subsequently, GenB3 catalyzes a series of transformations including dehydration at C-4′ and C-5′, dephosphorylation at C-3′, and the formation of a double bond between C-4′ and C-5′, to produce 6′-deamino-6′-oxosisonicin (6′-deamino-6′-oxoverdamicin) (Zhou et al., 2021; Li et al., 2021). The PLP-dependent aminotransferases GenB1, GenB2, GenB3, and GenB4 then catalyze transamination at the C-6′ position to form sisomicin (verdamicin C2a and verdamicin C2 in the parallel pathway). The bifunctional enzyme GenB4 catalyzes the final step of gentamicin di-deoxygenation through dual reduction and transamination. It performs a 4′,5′-reduction with the simultaneous oxidation at C-6′ on sisomicin (verdamicin C2a and verdamicin C2, respectively) by protonating the quinonoid intermediate at C-4′, thereby shifting the 4′,5′ double bond to the 5′,6′ position. Imine exchange subsequently releases an enamine, which is protonated at C-5′ to form an imine. Hydrolysis of this imine yields 6′-deamino-6′-oxogentamicin (C1a; C2a in the parallel pathway) (Li et al., 2021; Chen et al., 2020). Finally, transamination occurs again at the C-6′ position to generate the various gentamicin C components.

### 4.3 Underlying mechanisms and engineering of key enzymes

In-depth studies have been conducted on the multifunctional enzymes GenB3 and GenB4. Their amino acid sequences share 84.72% homology. In addition to their PLP-dependent transaminase activity, GenB3 also exhibits di-dehydroxylation activity, while GenB4 exhibits 4′,5′-double bond reduction activity. Crystallographic analysis and *in vitro* enzymatic assays have revealed that Ser57 is the key residue of the enzymatic activity of GenB3, whereas Asp52 is critical for that of GenB4. When these key residues were exchanged between the two enzymes, their dehydroxylation and double-bond reduction actions were also swapped (Li et al., 2021). Kong et al. used a semi-rational design method to mutate residues within 6 Å of the substrate-binding site of GenB3 to obtain three highly active mutants. The best-performing mutant, L361C, exhibited a nine-fold increase in activity compared to that of the wild-type enzyme. An analysis of the structure–activity relationship was performed using molecular dynamics simulations, which revealed that the L361C mutation induced rotation of the C-3′ phosphate group, which altered the hydrogen-bonding pattern between the key residue Ser57 and the substrate. This change stabilized the protein conformation and reduced the binding energy (Kong et al., 2024). In a similar approach, Zhai et al. used semi-rational design to enhance the activity of both GenB3 and GenB4. The GenB3 mutant M1 formed a hydrogen bond network within its active center, while the distance between K238 and the reaction center in the GenB4 mutant M1 was shortened (Zhai et al., 2025). These studies lay the foundation for the mild and efficient synthesis of C6′-modified aminoglycoside analogs. They also provide valuable insights into optimizing the di-deoxygenation process to synthesize greater levels of specific components in *M. echinospora* and offer new possibilities for engineering dideoxygenases in other aminoglycoside antibiotics.

## 5 Optimization of gentamicin components and construction of high-yielding strains

### 5.1 Overview of the industrial production of gentamicin

The industrial-scale production of gentamicin predominantly relies on submerged fermentation of *Micromonospora* spp. (e.g., *M. echinospora*, *M. purpurea*). Through decades of conventional mutagenesis and screening, industrial production strains have been optimized to achieve high antibiotic titers. The fermentation process is typically conducted in large-scale bioreactors, with stringent control of critical parameters including dissolved oxygen, pH, temperature, and carbon/nitrogen sources (e.g., glucose, starch, soybean meal powder) to maximize antibiotic yield. Following fermentation, the gentamicin C complex is isolated and purified from the culture broth through a series of downstream processing steps, including filtration, ion-exchange chromatography, solvent extraction, and crystallization (Xu et al., 2025). In recent years, metabolic engineering approaches have been successfully implemented to modify industrial strains, enabling targeted enhancement of specific components (e.g., gentamicin C1a) or the biosynthesis of novel derivatives. This advancement signifies a paradigm shift in gentamicin production—from traditional “black-box” fermentation toward a more precise and controllable “cell factory” model (Li et al., 2013; Wei et al., 2019).

### 5.2 Rational optimization and high-yield strain construction for gentamicin C1a and C2b components

Gentamicin C1a serves as the precursor for the semi-synthetic antibiotic etimicin (1-*N*-ethyl-gentamicin C1a) (Kumar et al., 2008). Etimicin is characterized by its high efficacy, safety, and broad antibacterial spectrum with low toxicity. It has demonstrated potent antibacterial activity against various clinically isolated drug-resistant bacteria, including Methicillin-Resistant *Staphylococcus aureus* (MRSA). To enhance gentamicin C1a production, Li et al. disrupted *gen*K, which encodes the C-6′ methyltransferase in *M. echinospora*. This genetically modified strain produced only two components, gentamicin C1a and C2b, with the proportion of gentamicin C1a increasing from 6.5 to 86.3% and a biological potency reaching 939.8 U/mL (Li et al., 2013). Building on this work, they further knocked out the 6′-*N*-methyltransferase gene *gen*L, which blocked the conversion of gentamicin C1a to C2b. This yielded a high-producing gentamicin C1a strain, which increased the yield of gentamicin C1a by 17.37% and achieved a biological potency of 1103.1 U/mL. In contrast, overexpression of *gen*L altered the ratio of gentamicin C2b to C1a from 2:7 to 5:3, increased the yield of gentamicin C2b by 263.11%, and produced a biological potency of 687.5 U/mL. In a complementary approach, Wei et al. simultaneously disrupted *genK* and *genL* in *Micromonospora purpurea*, engineering a high-yielding strain capable of exclusive production of the single component gentamicin C1a (Wei et al., 2019). Yang et al. reported that supplementing the fermentation medium with 0.1% CaCl_2_ and 0.3% sodium citrate increased the overall gentamicin potency by 11.5% and raised the proportion of C1a from 38 to 42% (Yang et al., 2023).

### 5.3 Construction of sisomicin high-producing strains via metabolic engineering

Sisomicin is a broad-spectrum aminoglycoside antibiotic with potent antibacterial activity against most Gram-positive and Gram-negative bacteria. The semi-synthetic aminoglycoside antibiotic netilmicin is derived from sisomicin by introducing an ethyl group at the N-1 position of the 2-DOS core. Netilmicin demonstrates low nephrotoxicity and ototoxicity, is insensitive to various bacterial inactivating enzymes, and remains efficacious against many gentamicin-resistant strains. By disrupting *gen*K and *gen*B4 in *M. echinospora*, a mutant strain was generated that produced only two components: sisomicin and G-52 (Chen et al., 2020). By further engineering the strain to knock out the 6′-*N*-methyltransferase gene *gen*L, the biosynthetic pathway from sisomicin to G-52 was blocked, resulting in the exclusive production of sisomicin. This increased the sisomicin yield by 3.41% and achieved a biological potency of 930.7 U/mL.

### 5.4 Efficient production of gentamicin B through a reconstituted glycosylation and modification pathway

In wild-type *M. echinospora*, the C molecules containing a C-2′ amino group are the main products, while gentamicin B, with a C-2′ hydroxyl group, is a naturally occurring minor component. Gentamicin B is the precursor of the semi-synthetic antibiotic isepamicin, which exhibits high stability against AMEs (Kumar et al., 2008). Capitalizing on the structural similarity of a unique C-2′ hydroxyl group in both gentamicin B and kanamycin A and integrating the 12-step biosynthetic pathway of gentamicin with the 2-step pathway of kanamycin, an artificial biosynthetic route was constructed that directly produced gentamicin B (Ni et al., 2016). First, the genes *gen*K and *gen*P in *M. echinospora* were disrupted to eliminate downstream metabolic pathways, which redirected the metabolic flux toward JI-20A accumulation. Subsequently, the two kanamycin biosynthetic genes *kan*J and *kan*K, which deaminate the 2′ position to generate a hydroxyl group, were introduced into the JI-20A-producing recombinant strain. The engineered pathway directly produced gentamicin B, thus demonstrating that artificial biosynthetic systems can be effectively designed to generate targeted aminoglycoside antibiotics. Since all gentamicin components are biosynthesized using UDP-GlcNAc and UDP-xylose as precursors, researchers sought to develop strains capable of channeling greater metabolic flux of these precursors toward gentamicin biosynthesis. The glycosyltransferases KanM1 and GenM2, responsible for the transfer of UDP-GlcNAc and UDP-xylose respectively, were overexpressed in the gentamicin B-producing strain *Micromonospora echinospora* JK4. Under appropriate glucose feeding conditions, gentamicin B production was enhanced by up to 54%. To validate the broad applicability of this approach, KanM1 and GenM2 were also overexpressed in a gentamicin C1a-producing strain, increasing the titer by 45%. These findings demonstrate that the overexpression of glycosyltransferases, which catalyze the transfer of primary metabolites into secondary metabolite biosynthesis, represents a viable strategy for enhancing gentamicin production (Wu et al., 2017). Furthermore, through *in vitro* experiments, Ban et al. demonstrated that gentamicin B components can be assembled via three independent biosynthetic pathways and identified seven novel intermediates (Ban et al., 2019). This finding provides detailed genetic and molecular insights for enhancing gentamicin B production. Furthermore, using *kan*J and *kan*K as probes, Chang et al. performed genomic mining on the gentamicin B-producing strain *Micromonospora echinospora* CCTCC M 2018898, identifying potential deamination-related genes *gen*R and *gen*S. Subsequent *in vivo* knockout and complementation experiments confirmed that *gen*R and *gen*S participate in the final step of gentamicin B biosynthesis (Chang et al., 2019). They overexpressed these two genes by placing them under the strong promoter kasOp* and *SRL37*, respectively, leading to a 64% increase of gentamicin B yield (798 mg/L) in the engineered strain.

Genetic manipulation of gentamicin-producing strains has enabled precise modifications of the biosynthetic pathway, leading to significant enhancement in the yield of specific components. These approaches have not only facilitated the creation of single-component producers but have also achieved remarkable production improvement. By leveraging genetic tools to finely regulate the production of key metabolites, metabolic engineering offers a promising avenue for further optimization (Xu et al., 2025).

## 6 Development of novel gentamicin-derived aminoglycoside antibiotics

First-generation aminoglycoside antibiotics such as gentamicin were once widely used in clinical practice. However, the subsequent emergence of drug resistance and adverse side effects caused significant concern. As research into the mechanisms of action and resistance of aminoglycosides deepened, particularly in conjunction with advances in ribosomal structure and studies on AMEs, the development of second- and third-generation semi-synthetic aminoglycoside derivatives gained momentum (Garneautsodikova and Labby, 2016). Examples of these include dibekacin, amikacin, and arbekacin derived from kanamycin, and isepamicin and netilmicin derived from gentamicin and sisomicin, all of which have been successfully marketed. Although the widespread use of quinolone antibiotics in the late 1970s led to a significant decline in aminoglycoside usage, aminoglycosides have recently regained attention in the medical community due to their ability to combat severe infections caused by multidrug-resistant bacteria (Park et al., 2017). Current research efforts are focused on developing fourth-generation aminoglycosides that block AME target sites and have reduced toxicity (Li and Sun, 2019).

### 6.1 Resistance mechanisms to aminoglycoside antibiotics

The primary mechanisms of resistance to gentamicin and other aminoglycoside antibiotics involve the structural modification of the antibiotic by AMEs and the alteration of the antibiotic-binding site on the 16S rRNA of the bacterial 30S ribosomal subunit through mutation or methylation. The amino and hydroxyl groups of aminoglycosides serve as hydrogen bond donors for a series of interactions with the bacterial ribosome; however, these same functional groups are also the targets of AMEs in pathogenic bacteria. Modification of these groups significantly reduces the affinity of aminoglycosides for the bacterial ribosome, thereby conferring resistance (Ramirez and Tolmasky, 2010). The currently identified inactivating enzymes are subclassified into three major groups: *N*-acetyltransferases (AACs), *O*-phosphotransferases (APHs), and *O*-nucleotidyltransferases (ANTs). Research by Noller et al. revealed that an A1408G mutation in the ribosomal A-site of 16S rRNA leads to a drastic reduction or even a complete loss of binding affinity for 2-DOS-containing aminoglycosides such as gentamicin. Alternatively, specific methylation of the A-site, mediated by bacterial 16S rRNA methyltransferases that modify nucleotides to m^7^G1405 or m^1^A1408, also confers high-level resistance (Doi and Arakawa, 2007). In recent years, plasmid-mediated 16S rRNA methyltransferases have been identified in diverse pathogenic bacteria. These enzymes confer high-level, broad-spectrum resistance to aminoglycosides by specifically methylating A1408 (Wachino et al., 2007; González-Zorn et al., 2005; Wachino et al., 2006). Collectively, these findings provide a robust foundation for the structure-based design and development of novel aminoglycoside derivatives with enhanced antibacterial potency and greater specificity for pathogenic RNA targets, as well as for the development of inhibitors targeting AMEs and 16S rRNA methyltransferases.

### 6.2 Biochemical mechanisms of aminoglycoside-induced toxicity and structure optimization strategies for toxicity mitigation

**Figure 11:**
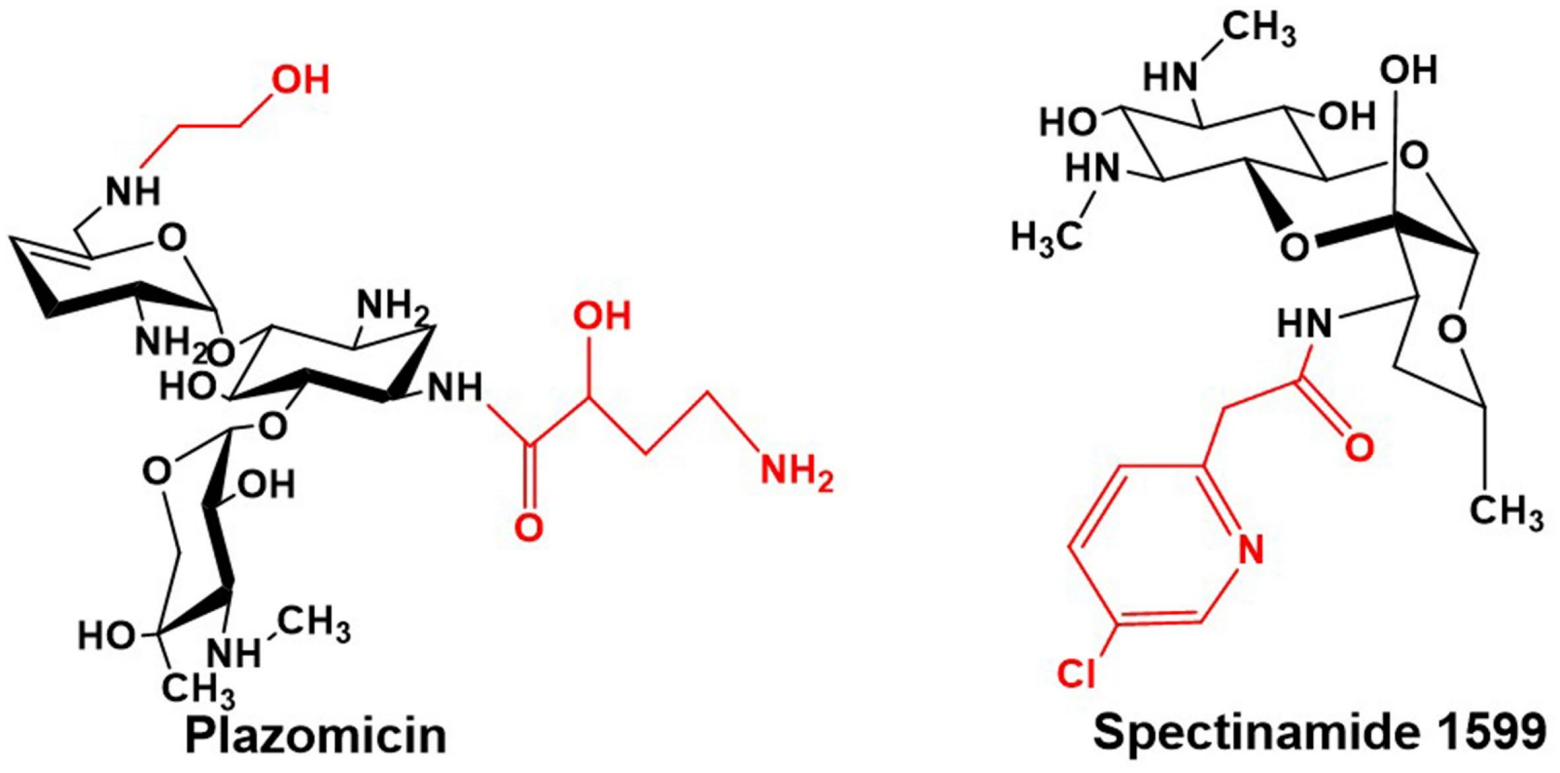
Structures of Plazomicin and Spectinamide 1599.

The nephrotoxicity and ototoxicity induced by gentamicin are primarily triggered by its accumulation in renal cortical and inner ear tissues. The underlying biochemical mechanisms involve complex interactions across multiple pathways (Lopez-Novoa et al., 2011; Le et al., 2023; McWilliam et al., 2020). A central event is drug-induced mitochondrial dysfunction, which mediates significant oxidative stress. This leads to excessive generation of reactive oxygen species (ROS), suppression of the Nrf2 antioxidant pathway, and reduced activity of enzymes such as SOD and GSH-Px, consequently initiating lipid peroxidation, malondialdehyde (MDA) accumulation, and direct damage to cellular structures (Quiros et al., 2011; Mahmoud, 2017; Jose et al., 2017). Concurrently, the non-specific binding of gentamicin to mammalian ribosomes further exacerbates mitochondrial dysfunction and promotes the apoptotic cascade. This process is particularly pronounced in individuals harboring mitochondrial rRNA mutations such as A1555G or C1494T (Hobbie et al., 2008; Ansari et al., 2016). Targeted structural modifications have proven effective in mitigating these toxicities. For instance, *N*-alkylation or hydroxyethylation at the C-6′ position (as seen in plazomicin, Figure 11) significantly reduces the drug’s affinity for the eukaryotic ribosomal decoding center via steric hindrance, thereby alleviating the inhibition of mitochondrial protein synthesis (Aggen et al., 2010). Conversely, deamination and hydroxylation at the C-2′ position (exemplified by isepamicin) not only diminish the initial interaction of the drug molecule with cell membranes but may also alter its charge distribution and intracellular trafficking. This ultimately weakens its accumulation propensity in target tissues and its capacity to induce oxidative stress (Kumar et al., 2008; Mahmoud, 2017). Collectively, these precise structural adjustments effectively control gentamicin-associated toxicity while preserving antibacterial activity. They operate by attenuating interactions with host ribosomes and reducing susceptibility to oxidative damage, thereby providing critical direction for the rational design of next-generation aminoglycosides.

### 6.3 Research and development of low-toxicity, antibiotic-resistant novel aminoglycoside derivatives based on structural modification

The recently developed semi-synthetic aminoglycoside antibiotic Plazomicin for anti-infective therapy is derived from sisomicin through chemical modifications that involve the addition of an AHBA side chain at the C-1 amino group and a hydroxyethyl side chain at the C-6′ amino group, thereby evading virtually all clinically relevant AMEs (Figure 11) (Armstrong and Miller, 2010). Plazomicin exhibits potent antibacterial activity against a broad spectrum of Gram-negative bacteria harboring diverse AMEs. It demonstrates particularly effective action against multidrug-resistant Enterobacteriaceae, including carbapenem-resistant strains (Haidar et al., 2016). This agent has now been approved for clinical use (Zhanel et al., 2012).

On the other hand, spectinomycin—a streptamine-containing aminoglycoside—serves as a second-line treatment for gonococcal infections, though its poor antimycobacterial activity has limited its utility in tuberculosis therapy. However, novel semi-synthetic spectinamide derivatives, such as spectinamide 1599, designed based on structural insights, selectively inhibit the ribosome and display remarkable activity against multidrug-resistant tuberculosis (Figure 11) (Lee et al., 2014). Both *in vitro* and *in vivo* studies confirm that chemical modification of spectinomycin effectively overcomes efflux pump-mediated resistance in *Mycobacterium tuberculosis*. Among these derivatives, spectinamide 1599 exhibits significant *in vivo* efficacy and a favorable safety profile *in vitro* (Viveiros and Pieroni, 2014), clearly underscoring the potential of structural optimization of classical aminoglycosides for the development of targeted anti-tuberculosis agents.

**Figure 12:**
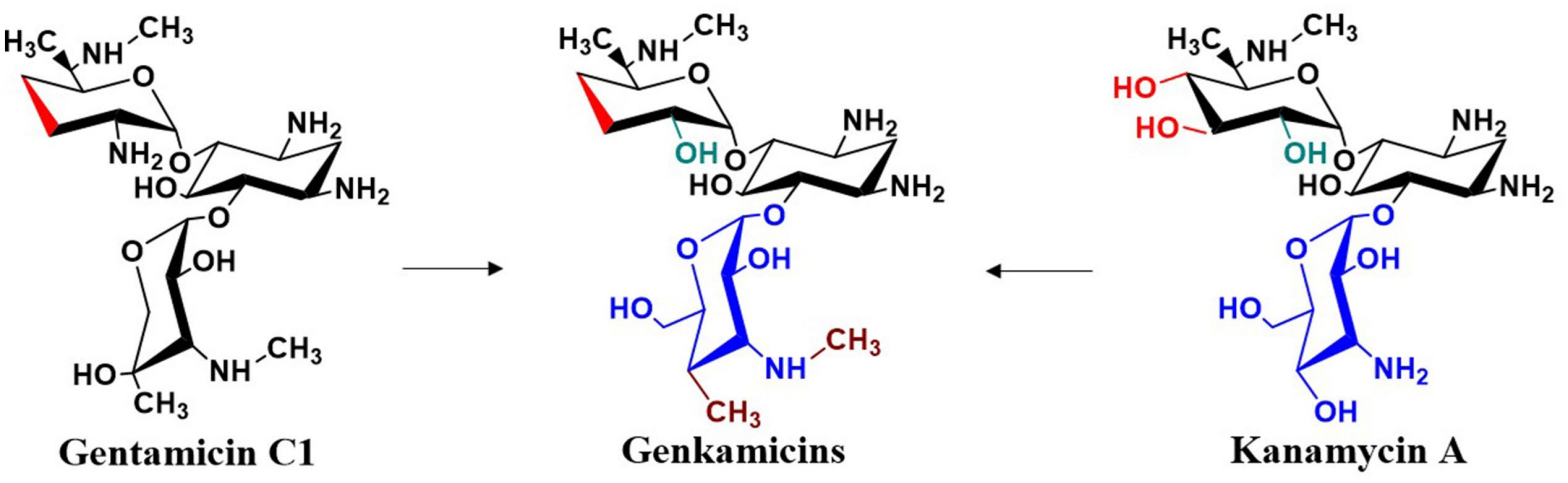
Structural modifications of gentamicin.

More recently, gentamicin and kanamycin have distinct advantages and limitations in their antibacterial activity, resistance profiles, and toxicity. Because their biosynthetic pathways have been largely elucidated, combinatorial biosynthesis strategies can be applied to intentionally or stochastically recombine and modify their biosynthetic gene clusters, or to incorporate genes encoding additional modifying enzymes. The goal of these modifications is to create novel aminoglycosides with reduced toxicity and enhanced activity against resistant strains compared to current aminoglycosides. Jian et al. replaced the glycosyltransferase GenM2 in the gentamicin pathway with KanM2 from the kanamycin pathway, producing a series of hybrid derivatives termed “genkamicins” (Figure 12). By regulating the hybrid biosynthetic pathway, six genkamicin molecules selectively accumulated that were subsequently isolated and identified. These compounds retained antimicrobial activity against a panel of World Health Organization critical priority pathogens. Notably, genkamicin-C2a demonstrated lower toxicity than clinical reference drugs in zebrafish embryos (Jian et al., 2024). In another study, Yang et al. introduced the glycosyltransferase KanM2 into *M. echinospora*, which replaced garosamine with kanosamine. The premature termination codon (PTC) readthrough activity of the resulting genkamicins was evaluated using a dual luciferase reporter assay. Among the examined genkamicins, genkamicin-Ae exhibited PTC readthrough activity comparable to that of the natural aminoglycoside G418, but with reduced toxicity. Moreover, genkamicin-Ae increased the expression levels of p53 and its downstream targets p21 and BAX, which promoted apoptosis in cancer cells (Yang et al., 2025). The use of combinatorial biosynthesis to develop gentamicin derivatives both expands the structural diversity of aminoglycoside antibiotics and provides promising directions for designing novel agents with low toxicity and high PTC readthrough activity.

While chemical synthesis played a predominant role in the early development of semi-synthetic aminoglycosides (e.g., amikacin, isepamicin), it frequently encounters challenges such as cumbersome steps, low yields, and environmental concerns during the construction of complex glycosidic bonds and the introduction of specific chiral centers. In contrast, biosynthetic engineering, particularly combinatorial biosynthesis and enzymatic catalysis, enables efficient and stereospecific assembly of the aminoglycoside core scaffold and complex saccharide moieties, demonstrating significant advantages in cost-effectiveness and green chemistry. For instance, engineered glycosyltransferases (e.g., GenM2, KanM2) facilitate the site-specific incorporation of sugar groups, thereby circumventing the tedious protection-deprotection strategies required in chemical synthesis (Jian et al., 2024; Yang et al., 2025). Prospectively, hybrid chemo-biocatalytic routes hold considerable promise: biosynthesis would be responsible for constructing the intricate chiral core and glycan backbone, while chemical synthesis could subsequently perform precise functional group modifications (e.g., *N*-alkylation, AHBA side chain introduction) on these advanced intermediates to efficiently generate novel derivatives with diverse structures. The successful development of plazomicin, which utilizes microbial fermentation to produce the sisomicin precursor followed by chemical modification, serves as a definitive exemplar of this strategy (Armstrong and Miller, 2010). This synergistic approach is anticipated to emerge as a mainstream paradigm for developing next-generation aminoglycoside antibiotics.

## 7 Conclusions and perspectives

This review provides a systematic overview of the complete biosynthetic pathway of gentamicin and recent research advances, with a particular focus on its unique methylation network, transamination–epimerization network, and di-deoxygenation processes. These intricate enzymatic networks not only establish a theoretical foundation for constructing engineered strains that overproduce specific components but also illuminate a path toward rationally designing novel, low-toxicity, and resistance-resistant aminoglycoside antibiotics through combinatorial biosynthesis strategies. Future research should concentrate on integrated approaches, including the rational design and directed evolution of key enzymes involved in di-deoxygenation (e.g., GenB3) and multifunctional enzymes (e.g., GenB2, GenB4) to alter their substrate specificity and catalyze the formation of novel “unnatural” derivatives. Concurrently, modular application of combinatorial biosynthesis—such as substituting the glycosyltransferase GenM2 in gentamicin with its counterpart from other aminoglycosides (e.g., KanM2 from kanamycin) to generate hybrid molecules (Jian et al., 2024), or introducing exogenous modification genes (e.g., *kan*J*-kan*K responsible for C-2′ deamination or genes involved in AHBA side chain synthesis)—would enable the direct biosynthesis of derivatives capable of evading inactivation by AMEs, such as C-2′ hydroxylated or *N*-acylated gentamicin analogs. Building on this, and inspired by successful cases like plazomicin, future work could focus on integrating multiple resistance-counteracting modifications (e.g., comprehensive di-deoxygenation and side-chain engineering) at common AME target sites (e.g., C-3′, C-4′, N-6′) on the gentamicin scaffold to develop next-generation agents effective against broad-spectrum resistance. Ultimately, establishing complementary high-throughput screening platforms that concurrently assess antibacterial activity (particularly against MDR clinical isolates), stability against AMEs, and low nephrotoxicity and ototoxicity will allow rapid identification of the most clinically promising candidates from a vast pool of engineered strains or derivatives, thereby systematically addressing the clinical challenges of resistance and toxicity associated with gentamicin. Furthermore, the mechanism of gentamicin transmembrane transport remains unelucidated and may represent a critical breakthrough for enhancing its industrial-scale production. Additionally, the regulatory mechanisms within the gentamicin biosynthetic pathway and the potential roles of non-coding regions constitute important future research directions for gentamicin and other aminoglycoside antibiotics.
